# Umbilical cord blood-derived non-hematopoietic stem cells retrieved and expanded on bone marrow-derived extracellular matrix display pluripotent characteristics

**DOI:** 10.1186/s13287-016-0437-6

**Published:** 2016-12-01

**Authors:** Junjie Wu, Yun Sun, Travis J. Block, Milos Marinkovic, Zhi-Liang Zhang, Richard Chen, Yixia Yin, Juquan Song, David D. Dean, Zhongding Lu, Xiao-Dong Chen

**Affiliations:** 1 Research Division, Department of Comprehensive Dentistry, University of Texas Health Science Center at San Antonio, San Antonio, TX 78229-3900 USA; 2 Department of Orthodontics, Fourth Military Medical University, School of Stomatology, Xi’an, Shaanxi Province 710032 People’s Republic of China; 3 Center for Reproductive Medicine, Ren-Ji Hospital, School of Medicine, Shanghai Jiao-Tong University, Shanghai, 200135 People’s Republic of China; 4 Department of Biomedical Engineering, University of Texas at San Antonio, San Antonio, TX 78249 USA; 5 Department of Plastic Surgery, Ren-Ji Hospital, Shanghai Jiao-Tong University School of Medicine, Shanghai, People’s Republic of China; 6 Biomedical Materials Engineering Research Center, Wuhan University of Technology, Wuhan, People’s Republic of China; 7 Department of Surgery, University of Texas Health Science Center at San Antonio, San Antonio, TX 78229-3900 USA; 8 Research Service, Audie L. Murphy Division, South Texas Veterans Health Care System, San Antonio, TX 78229-4404 USA

**Keywords:** Umbilical cord blood, CD146-positive cells, Plastic non-adherent cells, Extracellular matrix-adherent cells, Pluripotent stem cells, Extracellular matrix, Three germ layer tissue formation in vivo

## Abstract

**Background:**

Umbilical cord blood (UCB) not only contains hematopoietic stem cells (HSCs), but also non-hematopoietic stem cells (NHSCs) that are able to differentiate into a number of distinct cell types. Based on studies published to date, the frequency of NHSCs in UCB is believed to be very low. However, the isolation of these cells is primarily based on their adhesion to tissue culture plastic surfaces.

**Methods and results:**

In the current study, we demonstrate that this approach overlooks some of the extremely immature NHSCs because they lack the ability to adhere to plastic. Using a native extracellular matrix (ECM), produced by bone marrow (BM) stromal cells, the majority of the UCB-NHSCs attached within 4 h. The colony-forming unit fibroblast frequency of these cells was 1.5 × 10^4^/10^8^ mononuclear cells, which is at least 4000-fold greater than previously reported for UCB-NHSCs. The phenotype of these cells was fibroblast-like and different from those obtained by plastic adhesion; they formed embryonic body-like clusters that were OCT4-positive and expressed other human embryonic stem cell-related markers. Importantly, when implanted subcutaneously for 8 weeks into immunocompromised mice, these ECM-adherent and expanded NHSCs generated three germ layer-derived human tissues including muscle, fat, blood vessel, bone, gland, and nerve. Moreover, injection of these cells into muscle damaged by cryoinjury significantly accelerated muscle regeneration.

**Conclusions:**

These results indicate that UCB may be a virtually unlimited source of NHSCs when combined with isolation and expansion on ECM. NHSCs may be a practical alternative to embryonic stem cells for a number of therapeutic applications.

## Background

A practical and reliable source of pluripotent and/or multipotent stem cells holds the key to future stem cell therapies. Despite the great potential shown by human embryonic stem (hES) cells and induced pluripotent stem cells (iPSCs), there are a number of challenges that need to be overcome before they can be widely endorsed for use in large numbers of patients suffering from various aged-related diseases. These challenges include ethical concerns with regard to hES cells, potential long-term safety of transplanted iPSCs, and the considerable level of technical skill and cost associated with preparing both types of cells.

Umbilical cord blood (UCB) not only contains hematopoietic stem cells (HSCs) but also non-hematopoietic stem cells (NHSCs) that can differentiate into many distinct cell types including osteoblasts, chondrocytes, myocytes, endothelial cells, and neurons [1, 2]. Based on these findings, UCB has been proposed as an alternative source of mesenchymal stem cells (MSCs) for stem cell-based therapies [3, 4]. Compared to bone marrow (BM), a rich source of MSCs, UCB is widely available in abundance and can be harvested with little harm (i.e., low morbidity/mortality) to the donor [5, 6]. However, most studies report that the frequency of NHSCs in UCB is extremely low (~5 to 350 out of 10^9^ mononuclear cells (MNCs)) [6–9] and, compared to BM-derived MSCs (BM-MSCs), these cells are very difficult to grow in standard culture systems [10–12]. As a result, progress in developing UCB as a source of NHSCs for stem cell-based therapies has been largely thwarted.

Unlike HSCs, there are no reliable cell surface markers that specifically define MSCs or NHSCs. As a result, these cells are routinely obtained from BM and other tissues (e.g., UCB) using a standardized method based on adhesion to tissue culture plastic (TCP) [8, 13–15]. However, there is evidence that the non-adherent cell population contains a significant number of MSCs and the behavior of these cells is very different from those that adhere to TCP [16–18]. Based on these observations, we hypothesized that UCB contains an extremely immature population of NHSCs that have been previously overlooked because they fail to attach to TCP.

Previously, we reported on the production and characterization of a native extracellular matrix (ECM) generated by BM stromal cells [19, 20]. This ECM, deposited onto TCP dishes by the cells and subsequently decellularized, is 80–100 μm thick and displays a unique tissue-like architecture. It consists of more than 70 different matrix proteins including collagens, fibronectin, small leucine-rich proteoglycans, and several basement membrane components [19, 20]. MSCs cultured on this BM-derived ECM (BM-ECM) exhibit enhanced attachment, proliferation, and retention of stem cell properties, including differentiation into multiple cell lineages as well as the capacity for skeletogenesis [19–21].

The current study tests the above hypothesis and determines whether BM-ECM facilitates the efficient isolation of UCB-NHSCs by enhancing attachment and proliferation and promoting the retention of their stem cell properties.

## Methods

### Isolation and culture of NHSCs from human UCB

UCB was purchased from South Texas Blood & Tissue Center (San Antonio, TX, USA). MNCs were isolated from UCB using Ficoll-Paque Premium density media (GE Healthcare Bio-Sciences, Pittsburgh, PA, USA) as described previously [14]. MNCs were suspended in “growth media” containing alpha-minimal essential media (α-MEM; Life Technologies, Grand Island, NY, USA), penicillin (100 U/ml), streptomycin (100 μg/ml; Biofluids, Rockville, MD, USA), 15% pre-selected fetal bovine serum (FBS; Becton Dickinson, Franklin Lakes, NJ, USA), l-glutamine (4 mM), and basic fibroblast growth factor (bFGF; 4 ng/ml) and seeded at 10^6^ MNCs/cm^2^ onto TCP that was either uncoated or coated with fibronectin [22] or coated with ECM made by human BM stromal cells [19, 20]. After seeding, MNC cultures were incubated for 72 h at 37 °C to allow for attachment of the cells and then washed twice with phosphate-buffered saline (PBS) to remove non-adherent cells. After washing, growth media were added and half media changes were performed every 3 days. The adherent cells were cultured to 70–90% confluence in a humidified atmosphere at 37 °C. Adherent cells (passage (P)1) were detached from TCP surfaces using trypsin or the ECM surface using collagenase. An aliquot of the cells (P1) was used to determine the phenotype of the population using flow cytometry (see below), while the remaining cells were either used immediately or stored in liquid nitrogen. Over 100 UCB samples were obtained from the tissue bank and processed for this study. A subset of 50 of these samples was used in the current study because they had been more completely analyzed for cell phenotype.

### Human BM cells

Freshly isolated human BM-MNCs (containing MSCs), obtained from 20- to 30-year-old donors and purchased from ALLCELLS (Emeryville, CA, USA), were used to prepare BM stromal cell-derived ECM (BM-ECM) as described previously [20]. Previously, we demonstrated that BM-ECM is superior to uncoated TCP as a culture surface for human BM-MSCs and promotes attachment, expansion, and “stemness” of these cells [20]. For the present study, human BM-MNCs or MSCs were cultured on this ECM for comparison with UCB-derived cells.

### Flow cytometry

Anti-CD34, CD45, CD105, and CD146 antibodies were purchased from R&D Systems (Minneapolis, MN, USA). Anti-CD29 and SSEA-4 antibodies were purchased from BD Bioscience (San Jose, CA, USA). Single-cell suspensions (1–3 × 10^5^) were incubated for 30 min at 4 °C in 100 μl of specific antibody (10 μg/ml). Antibody-labeled cells were washed twice with staining buffer (PBS containing 5% fetal calf serum (FCS) and 0.01% sodium azide) and incubated in 20 μg/ml of FITC-conjugated goat anti-mouse IgG for 20 min at 4 °C. Cells were then washed two times with staining buffer and either analyzed immediately or fixed with 1% paraformaldehyde and analyzed within 96 h using a Becton Dickinson FACStar^plus^ (Franklin Lakes, NJ, USA) flow cytometer with 10,000 events collected per sample. The percentage of positive cells was determined by fluorescence-activated cell sorting (FACS). All assays were run in parallel using cells stained with isotype IgG as a negative control.

### Determination of cell population doubling time (PDT)

UCB-derived cells (P3–5), previously maintained on ECM, were seeded (4 × 10^3^ cells/cm^2^) onto ECM-coated plates in growth media and cultured for up to 9 days. At harvest, adherent cells were detached with collagenase and the number of live cells determined in triplicate. The average number of cells for each time point was plotted against time to generate a growth curve. PDT was determined as described previously [23].

### Immunohistochemistry

UCB-derived cells (P2), previously maintained on ECM, were seeded (2.5–7.5 × 10^4^ cells/cm^2^) onto chamber slides and cultured for 2 days in growth media. The cells were fixed with 4% formaldehyde for 30 min at room temperature, washed with PBS, and then blocked for 1 h using 5% normal goat serum containing 0.1% bovine serum albumin (BSA). The cells were stained by incubation with primary antibodies (1:100 dilution) against OCT4, TRA-1-60, and SSEA-4 for 30 min at 4 °C. Non-specific isotype rabbit IgG (1:100 dilution), mouse IgM (1:100 dilution), and mouse IgG (1:100 dilution) were used as negative controls, respectively. After washing with PBS, samples were incubated for 20 min with the appropriate FITC-conjugated secondary antibody. Specimens were mounted in DAPI-containing medium (Vector Laboratories, Burlingame, CA, USA) and the cells visualized using an Olympus FV500 Fluoview confocal microscope equipped with image analysis software to quantify fluorescence intensity (Olympus America, Inc., Melville, NY, USA).

### Determination of colony-forming unit fibroblast and osteoblast numbers

To determine the number of colony-forming unit fibroblast (CFU-F) colonies in the initial MNC population, freshly isolated UCB cells were seeded at low seeding density (1 × 10^5^ MNCs/cm^2^) onto ECM and uncoated or fibronectin-coated TCP surfaces and cultured at 37 °C in growth media. Half media changes were performed every 3 days. After 1 month, the cells were fixed and stained with crystal violet to determine CFU-F number. To assess CFU osteoblast (CFU-OB) colony formation, CFU-F colonies were maintained for an additional 25 days in osteoblast differentiation medium (growth media supplemented with 100 nM dexamethasone (Sigma), 50 μM l-ascorbate-2-phosphate (Wako Chemicals, Richmond, VA, USA), and 10 mM glycerol 2-phosphate). CFU-OB colonies were counted after staining with von Kossa.

### Adipogenesis and myogenesis

UCB-derived cells (P1–3), previously maintained on ECM, were seeded (4 × 10^3^ cells/cm^2^) onto ECM-coated plates in growth media and cultured to confluence. To study adipogenesis, the media were changed at confluence to differentiation media (DMEM containing 10% FBS, 0.5 mM IBMX, 1 μM dexamethasone, 10 μg/ml insulin, 100 μM indomethacin) [24] and one-half media changes were performed every 5 days. After 20 days in culture, adipocytes were visualized by staining with Oil Red O. To study myogenesis, the media were changed at confluence to differentiation media (DMEM containing 10% FBS, 5% horse serum, 10^-7^ M dexamethasone, 50 μM hydrocortisone) [24] and one-half media changes were performed every 5 days. After 25 days in culture, myotubes were identified after staining with hematoxylin and eosin (H&E).

### Cardiomyogenesis and angiogenesis

Cardiomyogenesis was induced as described previously [25]. Briefly, UCB-derived cells (P2) or BM-MSCs (P2), previously maintained on ECM, were seeded (10^6^ cells/cm^2^) onto TCP and cultured for 2 days in standard growth media, followed by an additional 2 days in media containing 5 μM 5-azacytidine (Sigma). Subsequently, the cells were switched to differentiation media (DMEM containing 10% FBS, 10^–4^ M ascorbic acid (Sigma) and 10 ng/ml transforming growth factor (TGF)-β) and cultured for up to 25 days with media replaced every 4 days. The expression of cardiac troponin T type 2 (TNNT2) was quantified using TaqMan polymerase chain reaction (PCR) after culture for 25 days in either growth or differentiation media.

To study angiogenesis, UCB-derived cells (P2), previously maintained on ECM, were seeded (3 × 10^3^ cells/cm^2^) onto six-well TCP plates coated with Matrigel™ (BD Bioscience, San Jose, CA, USA) in 1 ml endothelial differentiation media (EGM-2; Lonza, Switzerland) and cultured for 7 days at 37 °C. Media changes were performed every 3 days. Capillary formation was documented with brightfield photomicrographs. Baboon endothelial progenitor cells (positive controls) were kindly provided by Dr. Qiang Shi (TBRI, San Antonio). BM-MSCs (P2) were used as negative controls.

### Isolation of RNA and gene expression analyses using real-time PCR

Total RNA was extracted from cultured cells using Ultraspec™ RNA (Biotecx, Houston, TX, USA) [19]. RNA (2 μg) was reverse-transcribed using a High Capacity cDNA Archive Kit (Applied Biosystems, Foster City, CA, USA). The transcripts of interest, including housekeeping genes (GAPDH or β-actin), were amplified from the cDNA by real-time PCR using TaqMan Universal PCR Master Mix and TaqMan based primer and probe sets (TaqMan gene Expression assays, Applied Biosystems). Amplification and detection were carried out with an ABI Prism 7500 Sequence Detection System (Applied Biosystems) and gene expression quantified by subtracting the GAPDH threshold cycle (Ct) value from the Ct value of the gene of interest and expressed as 2^–ΔCt^. RNA isolated from hES (ESI6) was kindly provided by Dr. Peter J. Hornsby (UTHSCSA) and served as a positive control.

### In vivo implant assay

Cells (1 × 10^6^) were loaded onto Gelfoam (Pharmacia & Upjohn Company, MI, USA) or hydroxyapatite/tricalcium phosphate (HA/TCP) particles (Zimmer Inc, Warsaw, IN, USA) and implanted subcutaneously into the dorsal surface of 10-week-old immunodeficient beige mice (NIH-bg-nu-xid; Harlan Sprague Dawley, Indianapolis, IN, USA) [19] according to an approved IACUC protocol at UTHSCSA. After 8 weeks, implants were harvested, fixed, decalcified (HA/TCP implants only), and embedded in paraffin. Sequential sections were processed and stained with H&E. Bielschowsky’s silver stain was used to identify nerve [26]. To determine the origin of neotissues formed during implantation, a section adjacent to the H&E stained section was stained with an antibody specific for human nuclear ribonucleoprotein (Millipore, Billerica, MA, USA) [27].

### Green fluorescent protein labeling

To prepare ECM-adherent UCB cells stably expressing green fluorescent protein (GFP), commercially available Lentiviral particles (GeneCopoeia Lentifect™, LP-EGFP-LV105-0200) were used as described in the manufacturer’s instructions. Cells, labeled with an efficiency of >70%, were sorted using flow cytometry and GFP(+) cells (95% positive) were expanded for the experiments.

### Muscle regeneration using a cryoinjury animal model

A model of muscle cryoinjury was established in homozygous immunodeficient RAG2^–/–^, γc–/– mice (Taconic, Germantown, NY, USA) that were maintained as a breeding colony and housed in a temperature-controlled, air-filtered room with a 12-h light-dark cycle. All procedures were performed according to an approved IACUC protocol at UTHSCSA.

Adult mice (8 to 12 weeks old) were anesthetized and the gluteus maximus muscle exposed bilaterally. The oval shaped end (4 × 6 mm) of a 12 inch long copper probe was immersed in liquid nitrogen and then applied to the muscle for 20 s. Following cryoinjury, 10^6^ UCB-NHSCs were suspended in 35 μl 50% Matrigel™ (1:1 Matrigel™ to DMEM) and 10 μl aliquots of this suspension were implanted intramuscularly in three locations within the injured area, and then the skin was closed. Vehicle (35 μl 50% Matrigel™) and skin fibroblasts (10^6^ cells in 35 μl 50% Matrigel™) were used as controls. After 28 days, the animals were euthanized and muscle tissue harvested for histology or snap frozen in liquid nitrogen and stored at –80 °C.

### Statistical analysis

To successfully predict the isolation of NHSCs from UCB tissue, we initially employed a generalized linear regression model using MATLAB Statistics and Machine Learning Toolbox. The independent variables considered were: UCB sample volume, time since delivery in hours (time between tissue harvest and cell isolation in the laboratory), and cell surface phenotypic markers. The model indicated that all of the variables, except CD146, failed to identify a combination capable of predicting success. Next, we used a stepwise approach which excluded variables that failed to enhance our ability to predict the successful isolation of UCB cells. We began with the generalized linear regression model and then, in stepwise fashion, tested whether removing/adding variable(s) improved predictive power. The final model was arrived at by reaching a point where no single backward or forward step improved the model’s predictive accuracy.

All data are presented as the mean ± standard deviation (*n* = 3 or 6 depending on the experiment). All results were reproduced in at least six independent experiments with cells from six or more donors. For in vivo studies, a total of 18 implants, using cells from six or more donors, were performed. Statistical analyses were performed with the Student’s *t* test or one-way analysis of variance (ANOVA) with significance set at *p* < 0.05.

## Results

### MNC phenotype varies widely with UCB donor

**Fig. 1:**
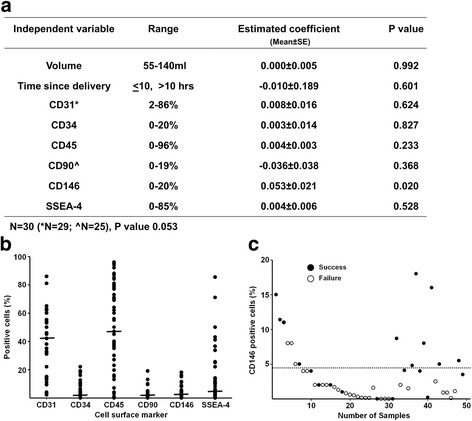
Recovery of NHSCs from UCB tissue varies widely from donor to donor, but CD146 expression is a valuable marker for predicting successful isolation of UCB-NHSCs. **a** Results of performing a general linear regression, using the independent variables shown, to predict the successful isolation of NHSCs from UCB. Although >50 UCB samples were evaluated, only 30 were analyzed for all of the listed independent variables; in the case of CD31 and CD90, only 29 and 25 UCB samples, respectively, were analyzed. CD146 was the only marker in the original MNC population capable of predicting the successful isolation of UCB-NHSCs (*p* < 0.02). **b** Cell surface phenotype (% positive cells) was highly variable in the MNC populations. More than 50 UCB samples were collected and analyzed. UCB samples were not analyzed for all cell surface markers, resulting in an unequal number of data points. The median is shown for each marker. **c** The percent of CD146-positive cells in the initial MNC population is a valuable marker for predicting successful isolation of NHSCs from UCB. There is a 50% likelihood of obtaining NHSCs when the initial population contains 4.33% CD146+ cells.

The expression of cell surface markers for HSCs (CD34), MNCs (CD45), endothelial cells (CD31), and MSCs (CD90, CD146, SSEA-4) was routinely measured upon arrival of UCB samples at the laboratory. As experience was gained in analyzing the samples, it became increasingly clear that this initial population of MNCs was very heterogeneous and displayed wide variation in all of the phenotypic markers examined (e.g., CD45: range 0–96%, median 45%; CD34: range 0–20%, median 2.2%) (Fig. 1a and b). Subsequently, after completing the analysis of all the samples (*n* = 50), it was observed that we had been successful at obtaining UCB-NHSCs in 35% of the samples. This led us to critically examine the data, focusing on those UCB samples where all markers had been measured, and look for potential attributes of the initial MNC population that increased the likelihood of successfully isolating UCB-NHSCs. The results of performing a generalized linear regression analysis indicated that only CD146 expression was likely to have a non-zero coefficient (*p* < 0.02) (Fig. 1a). Subsequently, a stepwise linear regression was performed and only CD146 expression was found to predict the successful isolation of UCB-NHSCs. Using the equation *p* = 0.06 (%CD146^+^) + 0.24 (*p* is the probability of success), there is a 50% likelihood of obtaining UCB-NHSCs, when the MNC population contains 4.33% CD146^+^, with 72% accuracy (Fig. 1c).

### UCB-derived colony-forming cells adhere to ECM but not TCP surfaces

**Fig. 2:**
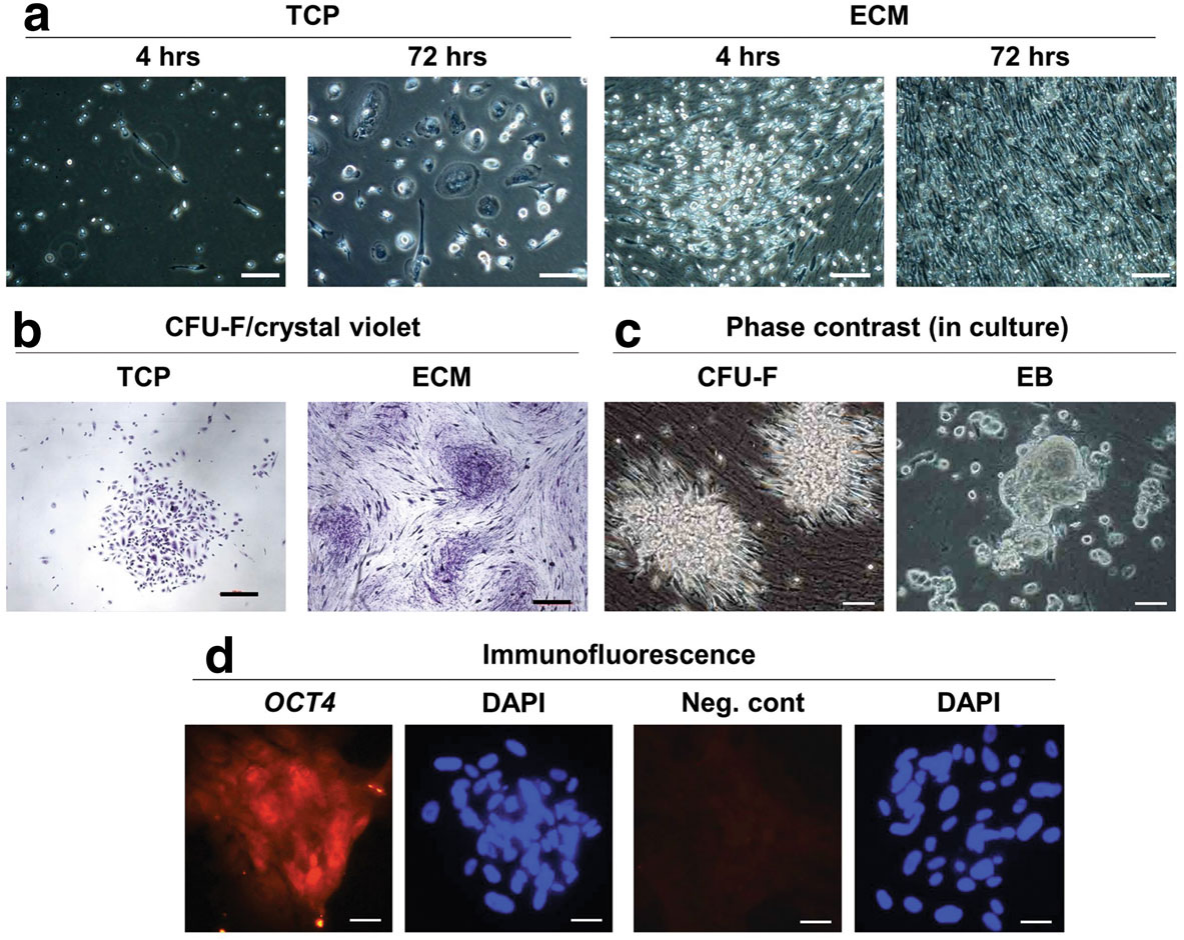
ECM promotes the attachment and CFU-F formation of UCB-NHSCs. **a** MNCs were isolated from UCB using a Ficoll-Paque Premium density gradient, seeded (1 × 10^6^ MNCs/cm^2^) onto either extracellular matrix (*ECM*) or uncoated tissue culture plastic (*TCP*), and incubated at 37 °C for up to 72 h as described in the Methods. At harvest, non-adherent cells were removed by washing with PBS and cell attachment to the surfaces recorded photographically under phase contrast microscopy. *Scale bar* = 100 μm. **b** Colony-forming units fibroblast (*CFU-F*) colony formation by primary (P0) cultures of MNCs on ECM or TCP surfaces. CFU-Fs were fixed and stained with crystal violet. *Scale bar* = 100 μm. **c** Phase contrast microscopy of CFU-Fs in culture. Some areas of the cultures have the appearance of embryonic bodies (*EB*). *Scale bar* = 10 μm. **d** OCT4, a marker used to identify ES cells, was expressed by EB formed by the UCB-NHSCs in culture. OCT4 was detected by immunofluorescence. Non-specific isotype IgG was used as a negative control (*Neg. cont*). Nuclear staining with DAPI is shown in *blue*. *Scale bar*  = 10 μm

To determine the effect of culture surface on retrieval of NHSCs, freshly isolated MNCs were plated onto TCP (with or without fibronectin) or ECM and incubated for 4–72 h. At the end of culture, media were removed and the cells washed with PBS to remove non-adherent cells. In Fig. 2a, it can be seen that an abundance of UCB-derived fibroblast-like cells attached to the ECM in as little as 4 h. With continued incubation (up to 72 h), cell density increased. In contrast, relatively few cells attached to TCP.

The frequency of NHSCs in UCB was measured using CFU-F assays. When MNCs were seeded on ECM at low density (10^5^ MNCs/cm^2^), the frequency of NHSCs was found to be approximately 1.5 × 10^4^ colonies/10^8^ MNCs. This is at least 4000-fold greater than reported by others (Fig. 2b) [6–9]. In addition, cells cultured on ECM generated embryonic body (EB)-like clusters that exhibited *OCT4*-positive staining, a unique feature of ES cells (Fig. 2c and d). None of these findings were observed on TCP.

**Fig. 3:**
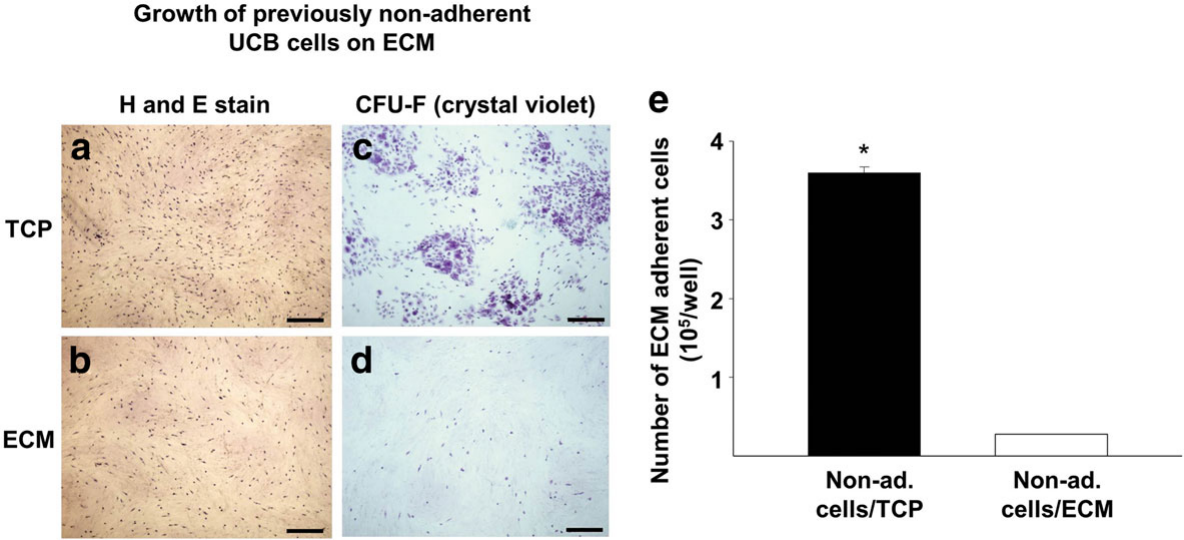
Large numbers of colony-forming cells can be recovered from cultures of TCP-non-adherent UCB-NHSCs by growth on ECM. Non-adherent (*Non-ad*.) UCB-NHSCs were collected from uncoated tissue culture plastic (*TCP*) or extracellular matrix (*ECM*) after 72 h of culture and then reseeded onto ECM plates. To visualize cell attachment to the ECM (after 24 h of culture), non-adherent cells were removed by washing with PBS and the remaining adherent cells stained with hematoxylin and eosin (*H and E*) (**a**, **b**). For colony-forming unit fibroblast (*CFU-F*) assay, the reseeded cells were cultured for 1 month on ECM, and then fixed and stained with crystal violet (**c**, **d**). *Scale bar* = 100 μm. **e** Number of UCB-NHSCs, previously non-adherent to uncoated TCP or ECM, collected after a second culture (rescued) on ECM for 24 h. *p < 0.05

To determine if NHSCs were present in the TCP-non-adherent population, we collected non-adherent cells from both surfaces and reseeded them onto ECM (Fig. 3). After 24 h, more of the TCP non-adherent cells were found attached to the ECM than previously non-adherent to the ECM (H&E stain) (Fig. 3a and b). Importantly, there was a 10-fold greater number of cells attached to the ECM, after originally being non-adherent to TCP, as compared to those previously non-adherent to the ECM (Fig. 3e). When culture on ECM was continued for CFU-F assay, the TCP-non-adherent cells produced ~10^4^ colonies/10^8^ MNCs (Fig. 3c) while the ECM-non-adherent group produced far less (Fig. 3d). Interestingly, the number of colonies generated by TCP-non-adherent cells, rescued on ECM, approximated that generated by cells initially seeded on ECM (1.0 × 10^4^ colonies/10^8^ MNCs vs. 1.5 × 10^4^ colonies/10^8^ MNCs, respectively). These results clearly suggest that UCB contains a large number of colony-forming cells that preferentially attach to ECM.

### ECM-adherent UCB-NHSCs display a pluripotent stem cell phenotype in vitro

**Fig. 4:**
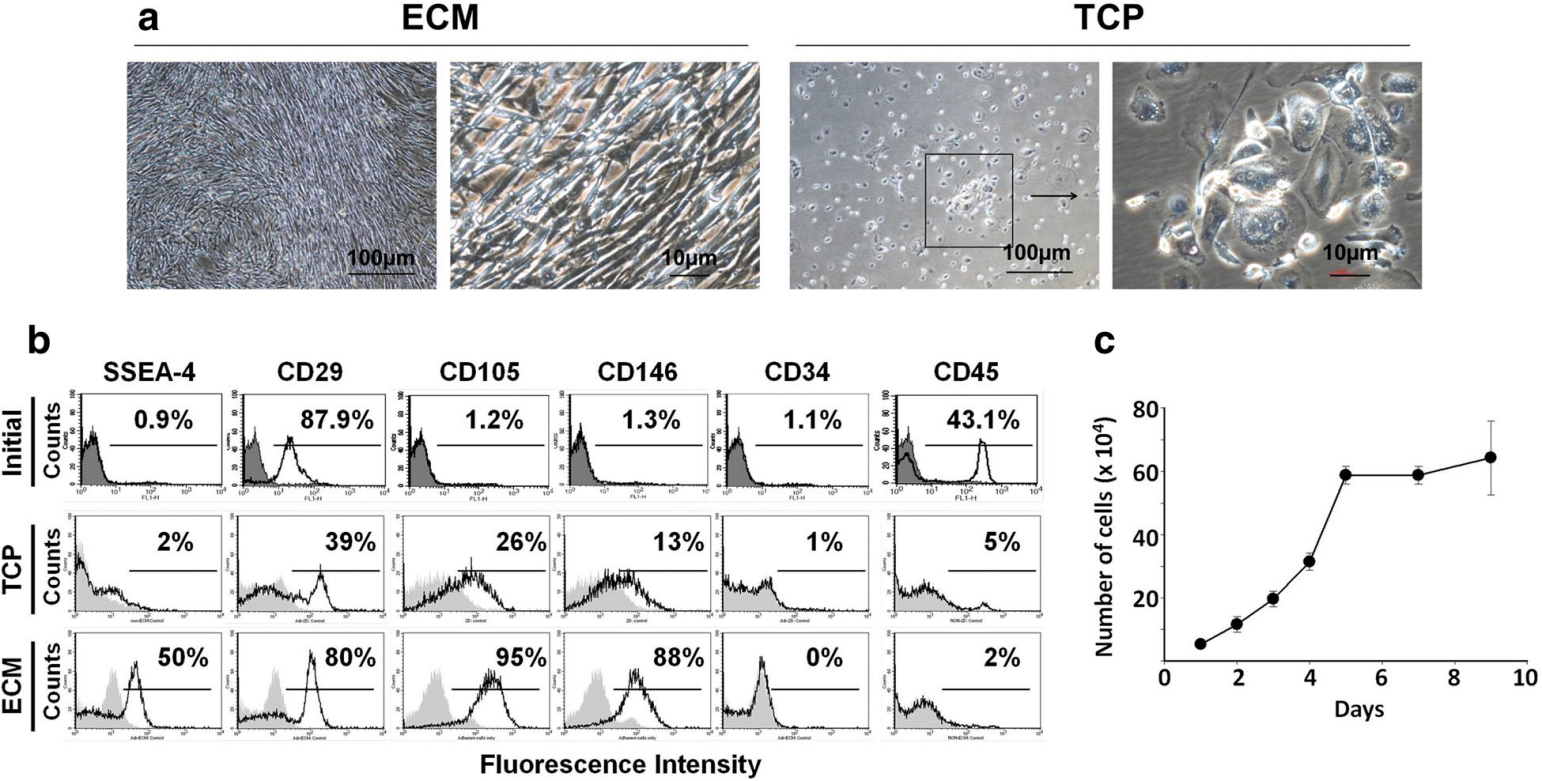
UCB-NHSC phenotype is different when grown on uncoated TCP versus ECM. **a** Culture on extracellular matrix (*ECM*) for 1 month promoted the growth of UCB-NHSCs better than uncoated tissue culture plastic (*TCP*). Phase contrast micrographs of cells cultured on the two culture surfaces are shown at two different magnifications. **b** Flow cytometric analysis of UCB-NHSCs grown on uncoated TCP or ECM. Cells grown on ECM expressed SSEA-4, an ES cell marker, as well as other MSC markers (CD29, CD105, and CD146), but not HSC markers (CD34 and CD45). Cells stained with non-specific antibody (isotype IgG) were used as negative controls (*gray* peaks). The initial population of MNCs from UCB was analyzed without culture and used for comparative purposes. **c** A growth curve of UCB-NHSCs maintained on ECM (P5). Population doubling time during the first 5 days of culture was determined as described in the Methods. Cell counts are expressed as the mean ± SD using triplicates

To determine the expansion capacity of NHSCs, cells were seeded at the same density on the different culture surfaces and grown for 1 month. At the end of culture, cells on ECM were confluent, fibroblast-like, and spindle-shaped (Fig. 4a). In contrast, cells on TCP (with or without fibronectin) grew poorly and failed to reach confluence. Cells that attached and grew on TCP were round, flat, and contained an abundance of cytoplasmic vacuoles suggestive of autophagy [28].

The phenotype of cells that grew on ECM was quite different from those on TCP. Expression of SSEA-4, a hES cell marker [29, 30], was found on 50% of the cells. In addition, a number of MSC-related markers, but not HSC markers, were expressed by 80–90% of the cells (Fig. 4b) [15]. The cells grew vigorously through 10 passages. P5 cells, shown in Fig. 4c, have a population doubling time of 29 h during the first 5 days in culture.

**Fig. 5:**
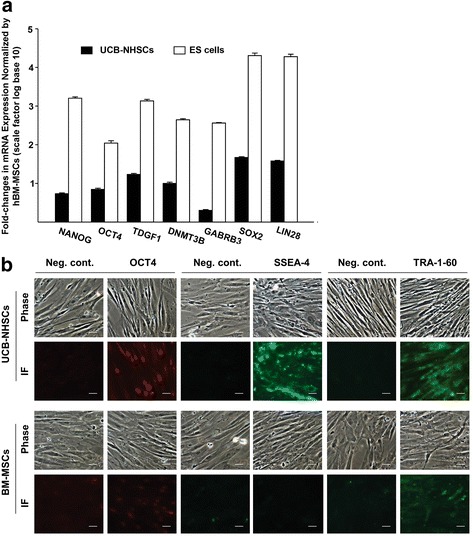
ECM-adherent UCB-NHSCs express ES cell markers. **a** The gene expression profile of P2 umbilical cord blood-derived non-hematopoietic stem cells (*UCB-NHSCs*) cultured on ECM was determined using TaqMan PCR. RNA isolated from human embryonic stem (*ES*) (ESI6) cells was used as a positive control, while RNA harvested from human bone marrow-derived mesenchymal cells (*hBM-MSCs*) was used for normalization of the data. All three types of cells were prepared, cultured on ECM, and harvested in parallel. The data shown represent the mean ± SD fold-changes (note: log base 10 scale on *y* axis) in transcript level after normalization to that of BM-MSCs. **b** Immunofluorescent (*IF*) detection of OCT4, SSEA-4, and TRA-1-60. UCB-NHSCs (P2) pre-cultured on ECM were seeded (2.5–7.5 × 10^4^ cells/cm^2^) onto regular chamber slides and cultured for 2 days. Cells were immunostained with antibodies specific for the proteins of interest. Non-specific isotype rabbit IgG, mouse IgG, and mouse IgM were used as negative controls (*Neg. cont.*) for OCT4, SSEA-4, and TRA-1-60, respectively. Parallel cultures of BM-MSCs were also prepared and treated similarly. Phase contrast micrographs of both types of cells are shown for comparative purposes. *Scale bar* = 10 μm

To further define the phenotype of NHSCs cultured on ECM, we examined genes strongly expressed by hES cells [30]. NHSCs were found to express *NANOG*, *OCT4*, *TDGF1*, *DNMT3B, GABRB3, Sox2*, and *LIN28* at 5-, 7-, 17-, 10-, 1.9-, 47- and 38-fold higher levels, respectively, than BM-MSCs obtained using the same ECM culture system (Fig. 5a). Unfortunately, it was impossible to obtain enough cells from TCP cultures to conduct the experiment. To confirm the expression of hES-associated genes by the NHSCs at the protein level, we immunostained cells using antibodies specific for OCT4, SSEA-4, and TRA-1-60 (Fig. 5b). In agreement with the gene expression data, NHSCs strongly expressed these ES cell-related markers as compared to BM-MSCs (Fig. 5b). These results suggest that UCB-NHSCs were a unique population of cells with characteristics of both MSCs and ES cells.

**Fig. 6:**
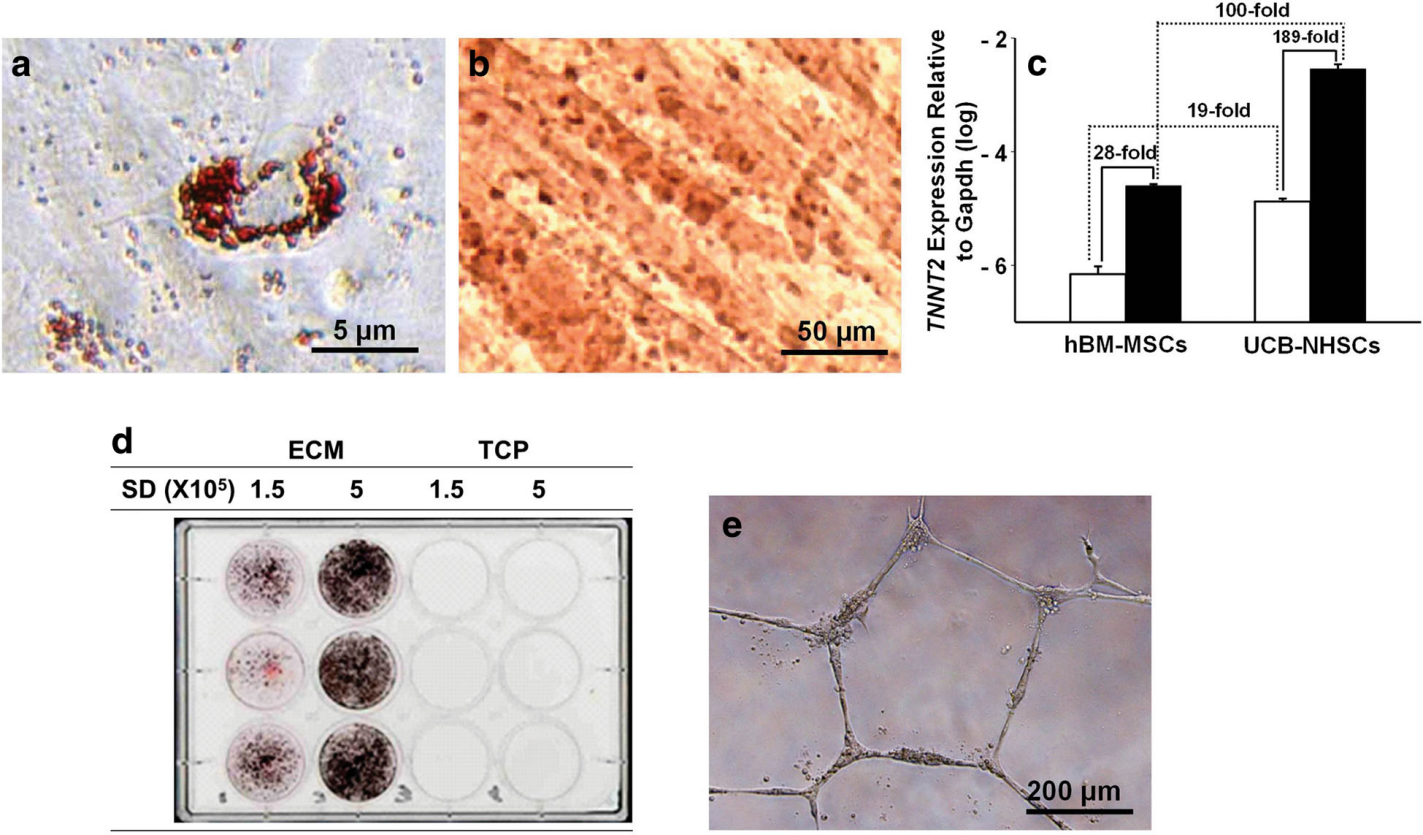
Extracellular matrix (*ECM*)-adherent umbilical cord blood-derived non hematopoietic stem cells (*UCB-NHSCs*) display the capacity for adipogenesis, myogenesis, osteogenesis, and angiogenesis in vitro. To examine differentiation capacity, UCB-NHSCs were seeded onto ECM or uncoated TCP plates and cultured to confluence. At confluence, the cultures were switched to differentiation media. **a** UCB cells cultured on ECM in adipocyte differentiation media formed Oil Red O-positive droplets. **b** UCB cells cultured on ECM in myogenic media formed myotubes containing multi-nucleated cells on the surface of the fibers. **c** UCB cells, cultured on ECM in regular growth media (*open bars*) or cardiomyogenic media (*solid bars*), expressed troponin T type 2 (*TNNT2*) and displayed enhanced expression in induction media. Similarly cultured human bone-marrow-derived mesenchymal stem cells (*hBM-MSCs*) displayed lower levels of TNNT2 expression compared to UCB cells, but also showed increased expression with induction media. **d** UCB cells, seeded at two different seeding densities (*SD*), were grown in osteogenic media on ECM and tissue culture plastic (*TCP*) culture surfaces. Von Kossa-positive mineral deposits were only observed with culture on ECM. **e** UCB cells cultured on Matrigel-coated wells developed capillary-like structures faster (48 h) than endothelial progenitor cells (positive controls, data not shown)

The differentiation capacity of UCB cells (P1 to P3) obtained by culture on ECM or TCP was examined by reseeding onto ECM or TCP and culturing to confluence. The cells were then switched to various differentiation media. UCB-NHSCs grown on ECM with adipogenic media differentiated into adipocytes (Fig. 6a). Similarly, cells in myogenic media differentiated and formed myotubes (Fig. 6b), while cells on TCP failed to grow. To test the cardiomyogenic potential of ECM-adherent UCB-NHSCs, confluent cells (P2) were maintained in differentiation media and the expression of TNNT2 [25] measured. The results showed that *TNNT2* expression by the differentiated cells was increased 189-fold compared to untreated controls (Fig. 6c). In contrast, *TNNT2* expression by BM-MSCs, similarly treated, was only increased 28-fold with induction. Interestingly, the basal level of *TNNT2* expression by ECM-adherent UCB-NHSCs was 19-fold higher than the BM-MSCs and in differentiation media the difference between the two types of cells was increased to 100-fold (Fig. 6c). Confluent NHSCs (P2) maintained on ECM in osteogenic media stained strongly with von Kossa (Fig. 6d). In contrast, no mineralization was observed with cells on TCP. To determine the angiogenic potential of the cells, UCB-NHSCs were seeded onto Matrigel-coated wells and evaluated for their ability to form microvasculature [31]. Surprisingly, UCB-NHSCs produced a capillary-like network within 48 h (Fig. 6e), which was significantly faster than observed with endothelial progenitors (positive controls) (data not shown).

### ECM-adherent UCB-NHSCs display a pluripotent phenotype and generate tissues in vivo

**Fig. 7:**
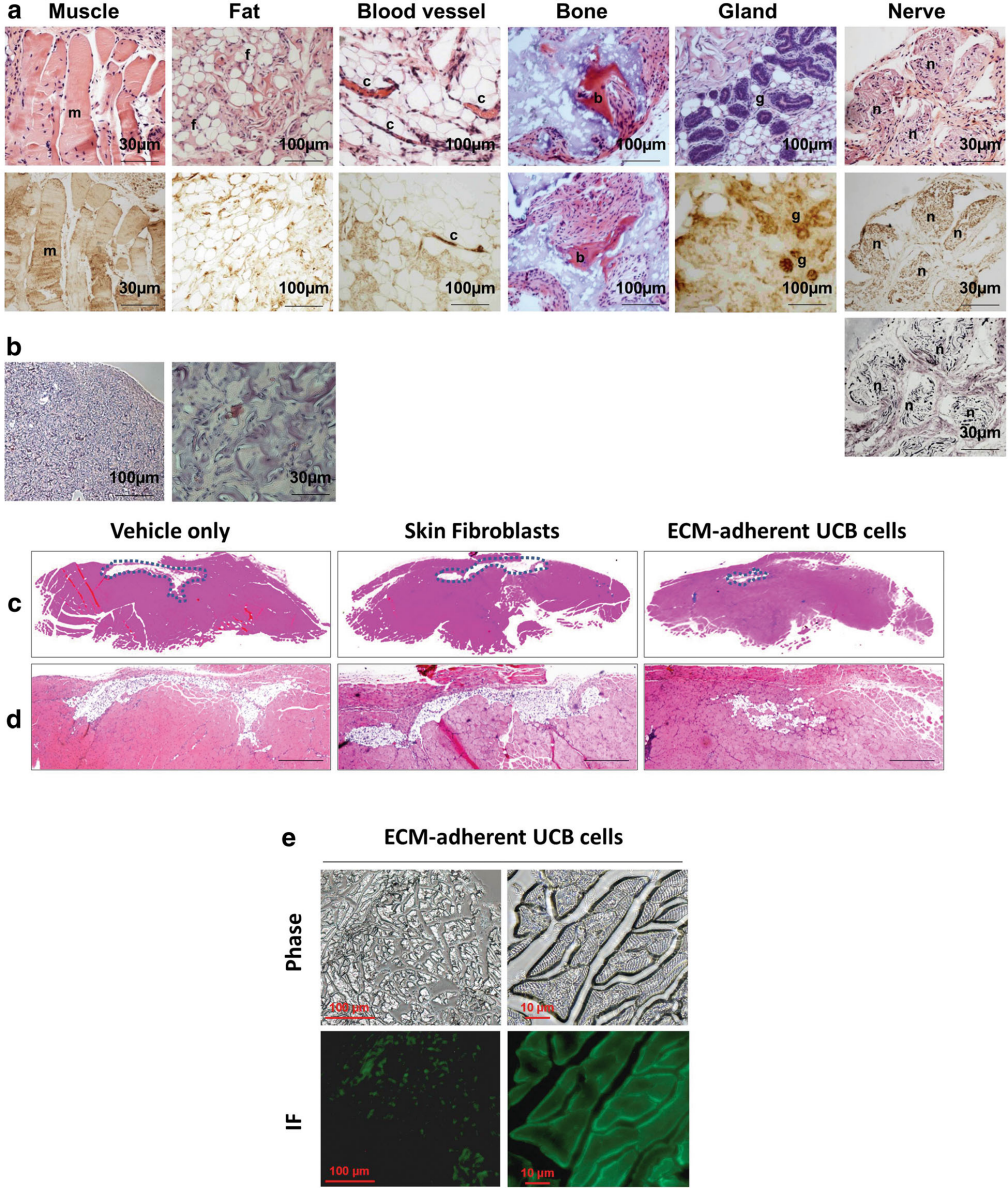
ECM-adherent UCB-NHSCs display a pluripotent phenotype and the capacity to regenerate skeletal muscle after cryoinjury. **a** ECM-adherent NHSCs were loaded onto Gelfoam or HA/TCP particles and implanted subcutaneously into immunodeficient mice. Implants were harvested after 8 weeks, processed for histological analysis, and stained with H&E (*upper panels*); Bielschowsky’s silver stain was used to identify nerve (see *lower panel* of “Nerve”) [26]. Sections were also immunostained for human nuclear ribonucleoprotein to confirm the origin of the cells responsible for neotissue formation (see *middle row* of panels except “bone”) [27]. Skeletal tissue (Bone) is only stained with H&E. Key to labels: *a*, artery; *b*, bone; *c*, capillary; *e*, endothelial cells; *f*, fat; *g*, gland; *m*, muscle; and *n*, nerve. **b** BM-MSCs were loaded onto Gelfoam carriers and implanted as controls. All procedures for experimental and control groups were performed at the same time. H&E stained sections demonstrated that BM-MSCs did not form heterogeneous tissues. **c** Histological cross sections of cryoinjured muscle tissue from immunodeficient mice treated with Matrigel alone (*vehicle*), Matrigel containing skin fibroblasts, or Matrigel containing extracellular matrix (*ECM*)-adherent umbilical cord blood (*UCB*) cells for 28 days. The perimeter of the wound is demarcated using *dark blue* dots. **d** Tissue morphology after H&E staining. UCB cells resulted in healing and a greater number of myogenic cells around the wound. *Scale bar* = 100 μm. **e** GFP-labelled UCB cells were injected into cryoinjured muscle to confirm that the cells were of human origin. Immunofluorescent (*IF*) staining demonstrated muscle regeneration by the labeled cells

To evaluate the ability of UCB-NHSCs to generate tissue in vivo, cells were loaded onto scaffolds and the cell-loaded scaffolds implanted subcutaneously in immunocompromised mice for 8 weeks. At harvest, the implants were processed for histology. Interestingly, implants loaded with UCB cells, obtained from the ECM culture system, generated multiple tissues originating from three germ layers (mesoderm: muscle, fat, blood vessel and bone; endoderm: gland; ectoderm: nerve) (Fig. 7a). To determine the cellular origin of the newly formed tissues, histological sections adjacent to those stained with H&E were immunostained with an antibody specific for human nuclear ribonucleoprotein [27]. Clearly, cells within the newly-formed tissues indicated their human origin (Fig. 7a, except for bone). Mouse and human tissues were used as negative and positive controls, respectively (data not shown). Skeletal tissue, found in ossicles within the implants, were of human origin [32]. Nerve tissue was specifically identified using Bielschowsky’s silver staining. In parallel, we also implanted scaffolds with or without BM-MSCs and found no heterogeneous tissue formation (Fig. 7b).

### ECM-adherent UCB-NHSCs have the capacity to regenerate skeletal muscle after cryoinjury

Based on studies showing that ECM-adherent UCB-NHSCs were able to generate muscle and blood vessels in vitro and after in vivo implantation, we tested the ability of these cells to regenerate skeletal muscle after cryoinjury. In this wound model, muscle injury is caused by touching a pre-cooled probe onto the gluteus maximus.

For the current experiments, ECM-adherent NHSCs were suspended in vehicle and then injected into each of three locations in the injured muscle. Another wound site was similarly injected with vehicle alone or skin fibroblasts as negative controls. After 28 days, the lesion area was measured on histological slides (Fig. 7c) and found to be decreased in wounds treated with NHSCs compared to vehicle- or skin fibroblast-treated controls. Although myogenic cells infiltrated the area around the edge of the wound, the number of myogenic cells (91 ± 7) was significantly higher in wounds injected with ECM-adherent cells compared to vehicle alone (72 ± 5) (Fig. 7d). Interestingly, instead of new muscle tissue, adipose tissue was observed in lesions treated with vehicle or skin fibroblasts. To identify the origin of myogenic cells in the newly-formed tissue, GFP-labeled UCB-NHSCs were injected into wound sites. The results clearly reveal that new muscle was produced by the implanted cells (Fig. 7e).

## Discussion

In the present study, we examined more than 50 UCB samples and achieved a 35% success rate of obtaining NHSCs which is similar to that reported by others [33, 34]. The data support the idea that these cells originated within the UCB and were not the result of “contamination” during collection. The relative lack of success in retrieving NHSCs may be explained by the large phenotypic variation found in the UCB samples and exemplified by the broad range of CD45 positive cells (0 to 96%; median, 45%) (Fig. 1a and b). The expression of this marker is especially informative since CD45 (“leukocyte common antigen”) is routinely used to define populations of MNCs from peripheral blood and BM and shows that these tissues are quite variable. To further assess the quality and variation of the UCB samples, we also examined the expression of CD34, a HSC marker. In the current study, the percentage of CD34-positive cells in the UCB MNCs was fairly low (range, 0 to 20%; median, 2.2%), but in the range previously reported for HSCs from peripheral blood and BM [35, 36].

Since UCB volume and “freshness” (i.e., time from delivery/parturition to cell isolation) have been suggested to predict the successful isolation of UCB cells [34], we included these two parameters, along with cell surface markers (Fig. 1a), in our general linear regression analysis to determine which combination of attributes more accurately predict success at retrieving NHSCs. It was found that CD146 expression was the only factor that correlated with success; the higher the percentage of CD146 in the population, the greater the chance of successfully isolating NHSCs. If CD146 expression reaches 4.33%, the chance of success will be 50%. Such a correlation may be explained by the fact that CD146 is a marker for pericytes which have been proposed to give rise to MSCs [37]. However, CD146-positive cells, isolated from the initial population of MNCs by cell sorting, failed to grow (data not shown). The fact that a high initial seeding density is required to stimulate NHSC growth suggests that other cells, such as MNCs, are needed. Nevertheless, the data indicate that in future studies it will be possible to increase our success rate by pre-selecting UCB samples with levels of CD146 expression above the threshold derived here (e.g., 4.33%).

One of the hypotheses of the current study is that the adhesion capacity of extremely immature NHSCs is poor. Indeed, our data strongly demonstrate that the majority of NHSCs fail to attach to TCP using classical methods for obtaining stem cells. In this study, NHSCs attached to ECM and grew rapidly for up to 10 passages, with a cell PDT of 29 h, when both the ECM and our modified expansion medium were provided. The NHSC population obtained using ECM was different from that obtained with TCP. The former consisted of a large number of stem cells that expressed SSEA-4, a hES cell marker, and MSC-related markers (e.g., CD29, CD105, and CD146) that were not found in the latter. It is widely accepted that attachment of primary NHSCs is a critical first step that must occur before they become activated. In our earlier studies, BM-MSCs grown on BM-ECM demonstrated both increased cell proliferation and retention of stem cell properties [19–21]. Here, the ECM-based cell culture system not only enriched the number of pluripotent stem cells obtained, but also facilitated the rapid expansion of these cells so that large quantities of high-quality stem cells could be recovered.

Interestingly, our results showed that the frequency of UCB-NHSCs was much higher than that of UCB-HSCs (1/10^4^–10^5^ MNCs). Since CD146 expression was the only factor that statistically correlated with successfully isolating UCB-NHSCs, it is likely that UCB-NHSCs are associated with pericytes located along the endothelial lining of capillaries.

To definitively evaluate stem cell self-renewal and pluripotency, in vivo studies are required. More specifically, it is essential to demonstrate that the cells spontaneously form multiple tissues from three germ layers in vivo without the use of differentiation-inducing agents [38]. Previously, “embryonic-like stem cells” from UCB were identified based on the expression of several markers associated with pluripotency without in vivo validation [14, 39]. However, marker expression, either at the mRNA or protein level, is not sufficient to demonstrate true stem cell pluripotency [40]. In particular, in vitro differentiation, induced by high concentrations of various reagents, is subject to artifact and a high level of false positives [41]. Our in vivo studies clearly indicate that UCB-NHSCs, obtained using the ECM culture system without inducing agents, were able to generate tissues from the three germ layers. Whether these heterogeneous tissues were generated from authentic pluripotent stem cells or lineage-committed cells remains to be clarified. Since the in vivo results showed that UCB-NHSCs generated enormous amounts of muscle and blood vessels, we further evaluated the capacity of these cells to repair skeletal muscle using a cryoinjury model. The results, using GFP-labeled UCB-NHSCs, showed that the majority of the newly-formed muscle was generated by the labeled NHSCs. In addition, the injected UCB-NHSCs may also recruit endogenous muscle progenitor cells to participate in tissue repair and this may explain the finding that some newly-formed muscle was not stained with GFP. Consistent with this idea, others have previously reported that transplanted MSCs have the capacity to re-activate endogenous stem cells in other organs [42, 43].

In our in vivo studies with NHSCs, teratoma formation was never observed. This may be explained by the modest expression of various ES cell-related genes, such as *LIN28* which is associated with the promotion of human cancers [44, 45]. In the present study, *LIN28* expression by ECM-adherent UCB-NHSCs was much lower than that of hES cells, even though it was about 37-fold higher than that of BM-MSCs. Ontologically, our findings suggest that NHSCs, obtained using the ECM, are closer to hES cells than BM-MSCs.

Although hES cells exhibit unlimited self-renewal and the capacity to differentiate into tissues from all three germ layers, the source (human embryos) of these cells raises ethical issues that restrict them from becoming a viable cell-based therapy. A number of the challenges associated with hES cells may be resolved by using iPSCs [46, 47]. However, iPSC technology is still in its infancy and there are many technical barriers to overcome before they can be used therapeutically [48, 49]. Here we report a new approach that enables the isolation of natural and highly multipotent NHSCs from an unlimited source, UCB, and that provides sufficient amounts of cells for systematic characterization. Given that the global birth rate is around 100 million per year [50], UCB remains the largest source of stem cells available. This virtually unlimited source of NHSCs makes them a viable alternative to hES cells for cell-based clinical applications. The information gained from this study will likely motivate the development of additional in vivo models for evaluating the use of NHSCs in various clinical applications.

## Conclusions

The results reported here support the existence of embryonic-like stem cells or highly primitive NHSCs in UCB [39, 51]. Importantly, the present study showed that: 1) the frequency of UCB-NHSCs is at least 4000-fold greater than that previously reported by others [6, 7, 9]; 2) ECM-adherent UCB-NHSCs form embryonic bodies that are OCT4-positive and express other hES cell-related markers; 3) UCB-NHSCs generate multiple tissues that originate from three embryonic germ layers in vivo; and 4) UCB-NHSCs accelerate skeletal muscle regeneration after cryoinjury. To the best of our knowledge, this is the first report that describes a method for successfully isolating substantial quantities of NHSCs from UCB and provides a characterization of these cells.

## Abbreviations

α-MEM: Alpha-minimal essential media
ANOVA: Analysis of variance
bFGF: Basic fibroblast growth factor
BM: Bone marrow
BM-ECM: Bone marrow-derived extracellular matrix
BM-MSC: Bone marrow-derived mesenchymal stem cell
CD: Cluster of differentiation
CFU: Colony-forming unit
CFU-F: Colony-forming unit fibroblast
CFU-OB: Colony-forming unit osteoblast
DMEM: Dulbecco’s modified Eagle medium
DNA: Deoxyribonucleic acid
ECM: Extracellular matrix
FACS: Fluorescence-activated cell sorting
FBS: Fetal bovine serum
FCS: Fetal calf serum
FITC: Fluorescein isothiocyanate
GAPDH: Glyceraldehyde-3-phosphate dehydrogenase
GFP: Green fluorescent protein
H&E: Hematoxylin and eosin
HA: Hydroxyapatite
HA/TCP: Hydroxyapatite/Tricalcium phosphate
hES: Human embryonic stem
HSC: Hematopoietic stem cell
iPSC: Induced pluripotent stem cell
MNC: Mononuclear cell
MSC: Mesenchymal stem cell
NHSC: Non-hematopoietic stem cell
P: Passage
PBS: Phosphate-buffered saline
PCR: Polymerase chain reaction
PDT: Population doubling time
RNA: Ribonucleic acid
Runx2: Runt-related transcription factor-2
SSEA-4: Stage-specific embryonic antigen-4
TCP: Tissue culture plastic (polystyrene)
TNNT2: Troponin T type 2
UCB: Umbilical cord blood
